# Association between CD4 T cell counts and the immune status among adult critically ill HIV-negative patients in intensive care units in Uganda

**DOI:** 10.12688/aasopenres.12925.1

**Published:** 2019-01-08

**Authors:** Arthur Kavuma Mwanje, Joseph Ejoku, Lameck Ssemogerere, Clare Lubulwa, Christine Namata, Arthur Kwizera, Agnes Wabule, Erasmus Okello, Samuel Kizito, Aggrey Lubikire, Cornelius Sendagire, Irene Andia Biraro

**Affiliations:** 1 Department of Anaesthesia, Makerere University, Kampala, 256, Uganda; 2 Department of Anaesthesia, Holy Cross Orthodox Hospital, Kampala, 256, Uganda; 3 Department of Anaesthesia, Uganda Heart Institute, Kampala, 256, Uganda; 4 Department of Anaesthesia, Mulago National Referral Hospital, Kampala, 256, Uganda; 5 Department of Clinical Epidemiology and Biostatistics, Makerere University, Kampala, 256, Uganda; 6 Medical Research Council, Uganda Virus Research-Institute Uganda Research Unit on AIDS, Kampala, 256, Uganda; 7 Department of Internal Medicine, Makerere University, Kampala, 256, Uganda

**Keywords:** CD4 T cells, HIV negative, critically ill, immune status

## Abstract

**Background**: Cluster of differentiation 4 (CD4) T cells play a central role in regulation of adaptive T cell-mediated immune responses. Low CD4 T cell counts are not routinely reported as a marker of immune deficiency among HIV-negative individuals, as is the norm among their HIV positive counterparts. Despite evidence of mortality rates as high as 40% among Ugandan critically ill HIV-negative patients, the use of CD4 T cell counts as a measure of the immune status has never been explored among this population. This study assessed the immune status of adult critically ill HIV-negative patients admitted to Ugandan intensive care units (ICUs) using CD4 T cell count as a surrogate marker.

**Methods:** A multicentre prospective cohort was conducted between 1 ^st^ August 2017 and 1 ^st^ March 2018 at four Ugandan ICUs. A total of 130 critically ill HIV negative patients were consecutively enrolled into the study. Data on sociodemographics, clinical characteristics, critical illness scores, CD4 T cell counts were obtained at baseline and mortality at day 28.

**Results:** The mean age of patients was 45± 18 years (mean±SD) and majority (60.8%) were male. After a 28-day follow up, 71 [54.6%, 95% CI (45.9-63.3)] were found to have CD4 counts less than 500 cells/mm³, which were not found to be significantly associated with mortality at day 28, OR (95%) 1 (0.4–2.4), p = 0.093. CD4 cell count receiver operator characteristic curve (ROC) area was 0.5195, comparable to APACHE II ROC area 0.5426 for predicting 24-hour mortality.

**Conclusions:** CD4 T cell counts were generally low among HIV-negative critically ill patients. Low CD4 T cells did not predict ICU mortality at day 28. CD4 T cell counts were not found to be inferior to APACHE II score in predicting 24 hour ICU mortality.

## Introduction

Cluster of differentiation 4 (CD4) is a glycoprotein found on the surface of immune cells such as T helper cells and macrophages ^1^. If CD4 T cells become depleted, the body is left susceptible to a wide spectrum of viral and bacterial infections that it would otherwise have been able to fight ^2^. CD4 T cells play a central role in the cascade of events forming immune response to foreign antigen, hence monitoring their levels is necessary to understand the extent of immune deficiency ^3^. A normal CD4 T cell count in an adult is usually between 500 and 1500 cells/mm³ ^4^. Low CD4 T cell levels are reported in HIV-positive patients as a marker of poor immune status and may fall to as low as zero cells in peripheral blood. Similarly, CD4 T cells may be suppressed among HIV negative patients that suffer from critical illnesses ^5^. CD4 T cell counts differ across different HIV-negative populations, due to a variety of factors that include environmental, immunological and genetic factors ^6^.

Critical care has become an important area of the health sciences, leading to development of scoring systems to guide clinicians in estimating patients’ prognoses, and in particular the risk of mortality. The most frequently used scoring system is the Acute Physiology Age and Chronic Health Evaluation II (APACHE II) ^7^ which predicts mortality in the first 24 hours of admission to ICU.

Low CD4 T cell counts were associated with mortality among HIV patients admitted to African ICU ^8^. Surprisingly, very low CD4 T cell counts are fairly common among people without HIV, and are likely to be present among 40 and 70% of people admitted to ICUs ^9^.

No such study had been conducted in Uganda before; hence, no available policies regarding use of CD4 T cell counts among critically ill HIV-negative patients from the Ugandan Ministry of Health.

## Methods

### Study background

We conducted a prospective cohort study between 1 ^st^ August 2017 and 1 ^st^ March 2018 at Mulago National Referral ICU, Uganda Heart Institute ICU, International Hospital Kampala ICU and Nakasero Hospital Limited ICU in Kampala city, Uganda. Baseline data on patients’ demographic variables (employment status, education level, family income, smoking, age, sex and ethnicity), admission diagnosis, CD4 T Cell counts and APACHE II scores were collected. We included adult HIV negative critically ill, APACHE II scored, medical/surgical ICU patients and excluded patients found admitted to ICU beyond 24 hours and those on immunosuppressant drugs such as steroids prior to admission. A total of 130 critically ill HIV-negative adults were enrolled into the study of which 127 participants gave written informed assent on behalf of their critically ill patients while 3 were waived of consent by the ethics committee because they had no proxies. The sample size was calculated using the formula for sample size calculation for two groups with a continuous outcome as outlined in Designing Clinical Research by Hulley *et al.* ^10^. ** We aimed for power of 80%, level of significance of 95% and using mean estimates of CD4 from a study ^6^. All study participants were followed for 28 days and end of follow up survival and mortality data was collected.

### Patient assessment

Referring to World Health Organization, we grouped CD4 levels into two; where CD4 above 500 cells/mm³ signified immune competent or normal CD4 count and those with CD4 less than 500 cells/mm³ reflecting low immunity.

The APACHE II scores and blood draws for CD4 T cell counts were performed upon admission between 8 am and 10 am. Blood sampling followed a standard laboratory practice. Approximately 3 to 5 ml of blood were collected in K3/K2 EDTA vacutainers, labeled with the patient’s identification, date and time of collection, and the name of the collecting personnel. To assess patients’ CD4 levels, BD FACSCalibur anticoagulated blood samples transported at ambient temperature (20–25°C) was stained within 48 hours of draw and then analyzed within 6 hours of staining ^11^. Samples were analysed from a 4-star laboratory of Makerere-Mbarara University Joint AIDS Program. Sample transport was by hand delivery and no transport was done on non-testing days. A coding manual for laboratory results was developed for broken samples, insufficient, clotted, frozen, haemolysed blood, samples not been drawn in K3/K2 EDTA vacutainers and errors in laboratory procedures. 

Strict procedures for data management during the pre-analytical, analytical and post analytical phase of testing were conducted to ensure the reliable production and delivery of accurate test results. Laboratory equipment was calibrated daily and sample laboratory registers were used to record receipt of samples and the production and release of results on entry of test result form.

The collection sites maintained the test request form. Testing laboratory had reliable systems for receiving and processing result data with uniform basic data handling, storage and reporting standards. The testing laboratory maintained records of result data for defined periods, to allow repeat reporting of lost test results, as well as aggregation for monitoring and evaluation or other research purposes. The testing laboratory also ensured reliable and rapid delivery of results.

### APACHE II questionnaire

The questionnaires were cross-checked by the principal investigator (PI) to ensure completeness before leaving the study site and periodically, the PI arranged a meeting with the assistants to validate data. Computer in-built checks reinforced data completeness. Quantitative data was double-entered to ensure correctness of data entered. According to WHO guidelines, the questionnaire was translated into Luganda a local dialect and back-translated into English by K.A.M.

To address potential sources of bias, the PI and critical care nurses (research assistants) sampled the participants by drawing blood and filling the questionnaires that were retained at the study sites. The laboratory technician (research assistant) transported all samples with only a laboratory request form and did not participate in drawing blood from the patients, only K.A.M. accessed the study results and strictly 130 participants were recruited and all completed a 28-day follow-up.

### Ethical approval

This study was approved by Research and Ethics Committee of Makerere University. A waiver of requirement for consent for unconscious patients without proxies was obtained with a reference number 2017-095. Final approval was granted by Uganda National Council for Science and Technology with a reference number HS104ES.

### Data management and statistical analysis

An electronic database was created using EpiData version 3.1 to enter the raw data from the questionnaires. The data was then transferred to STATA version 14.1 for analysis. In determining the CD4 T cell counts among the study participants, we presented the mean CD4 count with its corresponding standard deviation since it was normally distributed. In addition, we presented the CD4 as a categorized variable with frequencies (and percentages) for the various cutoffs with the corresponding 95% confidence intervals of the proportions.

In order to determine the relationship between CD4 T cell counts and 28-day ICU mortality, we performed multivariate logistic regression with CD4 count as the main predictor and 28-day mortality as the outcome. Prior to performing the multivariate logistic regression models, we performed bivariate analysis and all the variables with a p-value of 0.2 or less were included in the multivariate model.

Multivariate logistic regression was performed to determine how the CD4 jointly with other variables was associated with 28-day mortality. The variables were entered into a stepwise logistic model. Significance was set at p-value of 0.05 or less. The goodness of fit of the final model was tested using the Hosmer & Lemeshow goodness of fit, testing the null hypothesis that the final model adequately fits the data.

To assess the feasibility of using CD4 T cell counts to predict 24-hour mortality, as compared to APACHE II score, we compared the area under the Receiver Operator Characteristic Curves (ROC) between CD4 and APACHE II in predicting mortality. Prior to generating the ROC, we generated the sensitivities and specificities for the different cutoffs for both CD4 count and APACHE II. The ROC was then generated with y-axis being sensitivity and the x-axis being 1-specificity.

## Results

### Patient characteristics

More than half (53.9%) of the participants were recruited from MNRH followed by IHK (24.6%), NHL (19.2%) and lastly UHI (2.3%). Non-smoking self-employed black males dominated the study population at a mean age of 45.2±18.3 (mean±SD) and a family income above $1 as shown in Table 1. The major indication for admitting to ICU was postoperative high critical care requirements (46.2%), whilst the least common was urinary tract infection (UTI) (0.8%). Details are shown in Table 2. All raw data are available on OSF ^12^.

**Table 1.** Baseline demographic and clinical characteristics among critically ill HIV negative patients in Ugandan ICUs.

Variable	Patients, N (%) *
Hospital	
IHK	32 (24.6)
MNRH	70 (53.9)
NHL	25 (19.2)
UHI	3 (2.3)
Gender	
Male	79 (60.8)
Female	50 (38.5)
Age in years †	45±18
Ethnicity	
Black	122 (93.8)
Asian	3 (2.3)
Caucasian	2 (1.5)
Not disclosed	3 (2.3)
Family income	
Above $1 a day	65 (50)
Below $1 a day	59 (45.4)
Not disclosed	6 (4.6)
Employment status	
Professional Job	35 (26.9)
Self employed	60 (46.2)
Unemployed	31 (23.9)
Others	4 (3)
Education status	
University/tertiary	54 (41.5)
Secondary	33 (25.4)
Primary	34 (26.2)
None	5 (3.9)
Not disclosed	4 (3.1)
Smoking status	
Smoker	9 (6.9)
Non-smoker	115 (88.5)
Not disclosed	6 (4.6)
CD4 cell count time	
At 0800 h	90 (69.2)
At 1000 h	37 (28.5)
Others	3 (2.3)
Time to death (days) †	6.6±6.5
Status at 28 days	
Alive	93 (71.5)
Dead	37 (28.5)
Admission source	
Operating theatre	48 (36.9)
Medical wards	16 (12.3)
Obstetrics	2 (1.5)
Surgical wards	12 (9.2)
Private wing	3 (2.3)

*Unless indicated. †Data given as mean ± standard deviation.

**Table 2.** Showing indications for admission to ICU among critically ill HIV negative patients in Ugandan ICUs.

Variable	Patients, n (%)
Post-operative care	60 (46.2)
Central nervous system	
Stroke	10 (7.7)
Seizures	12 (9.2)
Head injury	29 (22.3)
Altered mental status (unknown cause)	14 (10.8)
Cervical spine injury	2 (1.5)
Other neurological indication ^1^	9 (6.9)
Cardiovascular	
Heart failure with cardiogenic shock	4 (3.1)
Post cardiac arrest	8 (6.2)
Acute MI	1 (0.8)
Others ^2^	5 (3.8)
Respiratory	
General respiratory distress	35 (26.9)
Severe pneumonia	8 (6.2)
Others ^3^	14 (10.8)
Gastrointestinal	
Gastro intestinal bleeding	6 (4.6)
Peritonitis	7 (5.4)
Other ^4^	5 (3.9)
Renal	
Acute renal failure	15 (11.5)
Infections	
CNS infections	3 (2.3)
Cardiac	2 (1.5)
Respiratory infections	19 (14.6)
Urinary tract infections	1 (0.8)
Gastrointestinal infections	7 (5.3)
Soft tissue infections	2 (1.5)
Blood stream	8 (6.2)
Sepsis	26 (20)
Malnutrition	6 (4.6)
Tumors ^5^	7 (5.4)
Trauma surgery	19 (14.6)
Scheduled surgery	18 (13.9)
Emergency surgery	16 (12.3)
Post-partum hemorrhage	3 (2.3)
Other indications	6 (4.6)
Comorbidities	12(9.2)

^1^Neurological diseases include brain tumors, cerebellar lesion. ^2^Cardiac diseases include arrhythmias, pericardial effusion and myoma. ^3^Respiratory diseases include aspiration pneumonia, bilateral pneumothorax, pulmonary embolism, pulmonary edema and other forms of chest trauma. ^4^Gastrointestinal diseases include intestinal obstruction, liver disease, cholelithiasis, and hepatitis, Other indications include hemorrhage, burst abdomen, drug toxicity, electrolyte imbalance, sick sinus syndrome. ^5^Include brain and lung tumors.

### CD4 T cell counts among critically ill HIV-negative patients

Overall 130 CD4 tests were carried out, of which 71 [54.6%, 95% CI (45.9-63.3)] were low (less than 500 cells/mm³). The mean CD4 count was 494.4±282 cells/mm³ (mean±SD), and the lowest count was 50 cells/mm³. Other details are shown in Table 3. There was no significant association in mortality outcome between those who had normal (CD4 ≥500 cells/mm³) and low (CD4 <500 cells/mm³) CD4 counts (p = 0.64). Other details are shown in Table 4.

**Table 3.** CD4 T cell counts among critically ill HIV-negative patients in Ugandan ICUs.

CD4 count, cells/mm ^3^	Patients, n (%)	95 % CI
Less than 100	4 (3.1)	0-6
100-499	67 (51.5)	42.8-60.3
500 and above	59 (45.4)	36.9-54.1

**Table 4.** Normal and low CD4 T cell counts among critically ill HIV negative patients in Ugandan ICUs.

Variable	CD4 count (cells/mm ^3^)		P value *
	Normal ≥ 500 (N=59)	Low < 500 (N=71)	
Age, years †	45.2±19.7	45.2±17.3	0.99
Outcome ‡			
Alive	41 (69.5)	52 (73.2)	0.64
Dead	18 (30.5)	19 (26.8)	
Time to death, days †	6.4±6.6	6.7±6.6	0.91
ICU stay (survivors) †	10.8±9.6	7.6±7.7	0.077

*For outcome, chi-squared test was used; for age, ICU stay and time to death, Student’s t-test was used. †Data given as mean±SD. ‡Data given as n (%).

### Relationship between CD4 T cell counts and 28-day mortality

At bivariate analysis, smoking, admitting a patient from another hospital, ICUs for hospitals MNRH, NHL and UHI had a strong statistically significant association with mortality at day 28. At multivariate analysis, abnormal CD4 count was not found to be significantly associated with mortality at day 28 in our population OR (95%) 1 (0.4–2.4) p = 0.093. Other details are shown in Table 5.

**Table 5.** Multivariate analysis for relationship between CD4 and 28-day mortality among critically ill HIV negative patients admitted to ICUs in Kampala.

Variable	28-day mortality, n/N (%)	aOR (95%)	P value
Age		0.2 (0-1.4)	0.093
CD4 count			
Normal (≥500 cells/mm ^3^)	18/59 (30.5)	1	
Low (<500 cells/mm ^3^)	19/71 (26.8)	1 (0.4-2.4)	0.990
Head injury			
No	25/101 (24.8)	1	
Yes	12/29 (41.4)	3.1 (1.1-8.8)	0.033
Sepsis			
No	27/104 (26)	1	
Yes	10/26 (38.5)	1.7 (0.6-5)	0.338
Gastrointestinal bleeding			
No	3/6 (50)	1	
Yes	3/6 (50)	3.7 (0.8-23.3)	0.167
Elective surgery			
No	36/112 (32.1)	1	
Yes	1/18 (5.6)	0.2 (0-1.4)	0.093
Admission source			
Operating theatre	10/48 (20.8)	1	
Medical wards	5/16 (31.3)		
Obstetrics	1/2 (50)	4.5 (0.2-85.1)	0.311
Surgical wards	4/12 (33.3)	1.2 (0.2-6.2)	0.830
A&E	12/42 (28.6)	0.8 (0.3-2.4)	0.716
Another hospital	5/16 (31.3)	8.5 (1.2-55.3)	0.026

aOR, adjusted odds ratio. In the model above, we adjusted for hospital, reasons for ICU admission, admission source and smoking history.

### Feasibility of using CD4 T cell counts to predict 24-hour mortality as compared to APACHE II score

**Figure 1.**
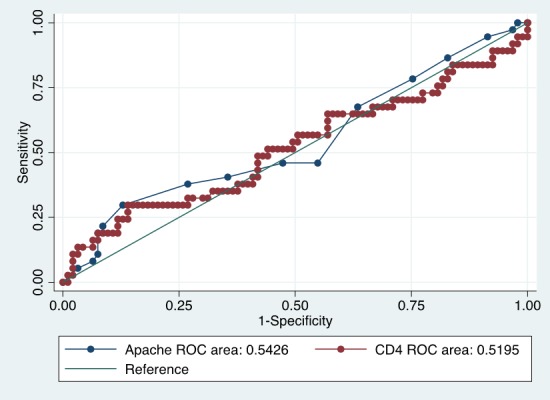
Receiver operator characteristic curve showing the feasibility of using CD4 cell counts to predict 24-hour mortality as compared to APACHE II score among critically ill HIV-negative patients.

From the receiver operator characteristic curves for comparing CD4 cell count and APACHE II score in predicting mortality, the area under the curve for the two graphs was comparable (this signified that CD4 count could be as good as APACHE II score). However, both graphs demonstrated very low area under the curve (the closer to 1 the area is, the more diagnostically accurate the curve). Therefore, the data signified that both APACHE II and CD4 were not good predictors of the outcome, despite being comparable ( Figure 1).

## Discussion

### Demographics and clinical characteristics

To our knowledge, this multicenter cohort study is the first report to discuss the immune status of critically ill HIV-negative patients admitted to Ugandan ICUs using CD4 T cell count as a surrogate marker. Almost all participants were black, of African descent and non-smokers, because black Africans, who rarely smoke, dominated the study population.

Most admissions from all the four ICUs were surgical cases and those requiring high postoperative care contributed the highest number of participants while the least was due to UTI. This is because MNRH is the referral center for most critical patients and strictly to mention the trauma patients. The same happened to UHI ICU that admitted mostly surgical cases.

In our study, we found that more than half of the participants had low CD4 T cell counts This may have been caused by critical illness that led to production of cortisol. This in turn may have suppressed the production of CD4 T cells. Our findings agree with a study conducted in nine consecutive patients admitted to the ICU with sepsis in Japan, whose CD4 cells were clearly reduced below 500 cells/mm³ and remained at that level for entire 4 weeks ^13^. These findings are also in agreement with a study conducted in HIV-negative Senegalese individuals, which found that CD4 cell counts varied in HIV-negative individuals ^6^. Though our study population was purely HIV negative, we found that more than 50% of the participants had low CD4 cell counts, with four participants having their CD4 cell counts as low as less than 50 cells/mm³ and six participants having counts less than 200 cells/mm³, values considered to indicate AIDS in patients living with HIV. Hence critical illness alone, without HIV infection, can present a picture that resembles that of AIDS in HIV-negative critically ill patients.

We did not find a statistically significant association between CD4 T cell counts and ICU mortality at day 28 among critically ill HIV-negative patients in our population. This is consistent with a study conducted by Feeney *et al*., which did not find whether low CD4 T cell counts were associated with a poor prognosis ^9^. The reason why this American study agrees with our findings could be entirely attributable to the sample size that is almost similar in both studies. However, our results contradict with other studies that have shown that septic patients with loss of CD4 T cells have a higher mortality ^14^. It is also in contrast with a study conducted in 2007, which showed that low CD4 T cell counts were associated with death ^14^. Our findings could be ascribed to the fact that CD4 T cells are a surrogate marker of the many immune cells. Hence, measuring CD4 alone could not yield reliable information to predict mortality. Another reason for the lack of statistical significance observed would be due to the sample size and short-term follow-up that may be were not adequate to give dependable results. It is also prudent to note that CD4 T cells were only sampled once, hence making it hard to track the exact CD4 cells at the time of the patient’s demise.

Both high APACHE II and low CD4 count could predict a 24-hour mortality in our population; however, despite being comparable, both were not good predictors of mortality. This is in line with a study conducted in 2000, where elevated APACHE II score remained a significantly negative predictor of survival at 28-day mortality ^15^. It also concurs with a study conducted in 2015 that reported that the median APACHE II of 25 predicted greater than 50% mortality ^8^. The latter leaves a benefit of doubt, as the study did not report that mortality would be 100%. However it is in contrast with a study done in 1995 that did not find any relationship between CD4 counts and APACHE II score, predicted mortality rate, or survival rate ^9^.

## Conclusion

From our study, we conclude that CD4 T cell counts were generally low among HIV-negative critically ill patients and recommend that this indicator should be incorporated onto the panel of baseline investigations in this group of patients. We also established that low CD4 cells did not predict mortality at day 28 in our study population, although it would predict 24-hour mortality and was not inferior to prediction using APACHE II score. Hence, we suggest the use of CD4 T Cell counts in resource constrained setup to help in directing proper use of resources. Critically ill patients with low CD4 T cell counts should be supplemented with immunoadjuvant therapy to restore their immune system and also prevent loss of functional T helper cells as these play a major role in defending the body against pathogens. Further multinational studies on serial CD4 sampling until patients’ demise and a longer follow-up period are required.

## Data availability

Raw data associated with this study are available on OSF in csv and dta formats. DOI: https://doi.org/10.17605/OSF.IO/JBMKP ^12^.

Data are available under the terms of the Creative Commons Zero "No rights reserved" data waiver (CC0 1.0 Public domain dedication).
