# Supplementary material for: Association between CD4 T cell counts and the immune status among adult critically ill HIV-negative patients in intensive care units in Uganda
Source: AAS Open Res. 2019 Jan 8;2:2. [Version 1] doi: 10.12688/aasopenres.12925.1 (PMC6742509; doi:10.12688/aasopenres.12925.1)
Supplement: Peer review history

# Peer review: Reviewer response for version 1

**DOI:** 10.21956/aasopenres.13997.r26948

**Published:** 2019-07-05

**Authors:** Djibril Wade

**Affiliations:** 1 IRESSEF (Institute of Research in Health, Epidemiological Surveillance and Training), Dakar, Senegal

The article is well written and easily understandable. It deals with the use of CD4 T-cell counts as a surrogate marker for 28 days mortality in HIV negative patients which is really interesting and will bring a new usage of the CD4 T-cell count which was mainly used to monitor immune system monitoring in HIV-infected patients.

 I would just recommend adding reference to the studies that established the normal values of CD4 T-cell counts in the Ugandan population (Nanzigu *et al.*, 2011 ^1^) where the 95% reference ranges for absolute CD4 count was 418 - 2105 cells/µL. In some countries mainly in resource-limited settings, people are exposed to a variety of infectious diseases and other conditions including stress that may affect CD4 count, and this is highly expected in patients attending ICUs. Given the normal values of CD4 counts in Uganda, my final recommendation will be to please adjust what you considered low CD4 counts.

I confirm that I have read this submission and believe that I have an appropriate level of expertise to confirm that it is of an acceptable scientific standard, however I have significant reservations, as outlined above.

## References

[1] : CD4-T-Lymphocyte Reference Ranges in Uganda and Its Influencing Factors. *Laboratory Medicine*.2011;42(2) : 10.1309/LMFT0VCE1UGO9YGD 94-101 10.1309/LMFT0VCE1UGO9YGD

# Peer review: Reviewer response for version 1

**DOI:** 10.21956/aasopenres.13997.r26831

**Published:** 2019-04-16

**Authors:** Banson Barugahare

**Affiliations:** 1 Faculty of Science and Education, Busitema University, Tororo, Uganda

I have reviewed the manuscript *“Association between CD4 T cell counts and the immune status among adult critically ill HIV-negative patients in intensive care units in Uganda”. *The finding that CD4 T cell counts were generally low among HIV-negative critically ill patients but did not predict ICU mortality is fundamental. This result calls for further immunological studies. Nevertheless, I would like to recommend the authors to review and make reference to the previous Ugandan population based CD4 normal value studies. This information is available from a couple of studies by Tugume *et al.* (1995 ^1^) and Lugada *et al.* (2004 ^2^). The background literature from these papers will inform the discussion and conclusion of this study to a more acceptable position than it is now.

I confirm that I have read this submission and believe that I have an appropriate level of expertise to confirm that it is of an acceptable scientific standard.

## References

[1] : Hematological reference ranges among healthy Ugandans. *Clin Diagn Lab Immunol*.1995;2(2) :233-5 769753510.1128/cdli.2.2.233-235.1995PMC170134

[2] : Population-based hematologic and immunologic reference values for a healthy Ugandan population. *Clin Diagn Lab Immunol*.2004;11(1) :29-34 1471554110.1128/CDLI.11.1.29-34.2004PMC321349

# Peer review: Reviewer response for version 1

**DOI:** 10.21956/aasopenres.13997.r26753

**Published:** 2019-02-26

**Authors:** Martin W. Dünser

**Affiliations:** 1 Kepler University Hospital, Linz, Austria

This is a very interesting work examining a novel aspect of intensive care medicine. The authors need to be commended for their efforts. Despite several positive aspects, I have some concerns and comments regarding the presented manuscript. Comments are enumerated in the order they appear in the text:

  

1. I suggest that the authors include a separate “Inclusion/Exclusion criteria” paragraph into the Methods section. In this paragraph, they need to be clear about the criteria used. 
2. One of, if not the most, important comments is that the authors explain how they determined negative HIV-status. Was this always done by negative HIV testing? Which test was used? Please give specific details about this as it is key for your argumentation that you included only HIV negative patients. 
3. Methods section, Study background paragraph: Please re-phrase the sentence that participants gave written informed consent on behalf of their critically ill patients. Did you mean that the next of kin gave written informed consent on behalf of their critically ill relatives? 
4. It is unclear how the authors performed a sample size calculation for two groups if they designed a cohort study which included only one arm. This is not possible. Please clarify or omit. 
5. Please clearly describe your primary and secondary study endpoint in the Methods section (e.g. the Statistical Analysis paragraph). 
6. Table 1: In the text it is given that the CD4 count was determined between 8 and 10 am. In Table 1 it is given that the CD4 count was measured at 8 or 10 am. Please clarify. After all, why is it important whether CD4 count is determined at 8 or 10 am? 
7. Table 2: Please indicate n (%) for all summary diagnoses (e.g. Central nervous system, cardiovascular, respiratory, etc.). Furthermore, please specify 'general respiratory distress' (which is a very broad term) and 'cardiac'. 
8. Results, page 5, first paragraph, last one before sentence: Please re-phrase as it is difficult to read. 
9. Results, page 5, subheading “Relationship between […]”. The paragraph suggests that you tested an association (multivariate analysis) and not a relationship. 
10. Table 3: What do you think about changing Table 3 into a scatterblot figure?
11. Table 5: Did you include age as a binary variable? Why is the OR below one for age? The higher the age the lower the mortality? This does not make sense. The OR (CI95%) for medical wards is not given. 
12. Since CD4 count was only determined at a single time point after ICU admission, it is unclear how the dynamics of this parameter is. Do you have data to analyse the association between CD4 count and the duration of hospital stay or disease duration before ICU admission?  
13. Figure 1 simply shows that both APACHE II and the CD4 count are useless for prediction of 24-hour mortality. This could be mentioned in the text but does not deserve presentation in a figure. Please omit. Moreover, prediction of 24-hour mortality is not a common goal in intensive care medicine. From the point of view of this reviewer, APACHE II is not validated to predict mortality at this early stage of critical illness. 
14. The limitation that this is a single centre study and that it is unclear whether its results can be extrapolated to other centres and regions should be mentioned. 
15. The conclusion paragraph of the text is too low and partly not supported by the results of the study. Please rephrase the conclusion paragraph of BOTH the abstract and the main text to: “In this HIV-negative critically ill population, CD4 count was <500 cells/mm³ in 51.5% of patients. We found no association between the CD4 count and mortality at day 28.” 
16. Overall, the manuscript would benefit from proof reading by a native English Speaker.

I confirm that I have read this submission and believe that I have an appropriate level of expertise to confirm that it is of an acceptable scientific standard, however I have significant reservations, as outlined above.
